# An Abstract of the Symptoms

**Published:** 1892-02

**Authors:** 


An Abstract of the Symptoms, with the latest dietetic and medicinal treatment of various diseased conditions. New York, Reed & Carnrick, 447 and 449 Greenwich St., 1891.

This small volume gives the latest views in relation to digestion and assimilation and their diseases, with the treatment of the same from the standpoint of the regular school of medicine. The treatment recommended, however, is with the elegant specialties made by Messrs. Reed & Carnrick, whose advertisement appears on advertising page 3 of this number of the journal.

